# Initial Immunopathogenesis of Multiple Sclerosis: Innate Immune Response

**DOI:** 10.1155/2013/413465

**Published:** 2013-09-24

**Authors:** Norma Y. Hernández-Pedro, Guillermo Espinosa-Ramirez, Verónica Pérez de la Cruz, Benjamín Pineda, Julio Sotelo

**Affiliations:** ^1^Neuroimmunology and Neuro-Oncology Unit, Instituto Nacional de Neurología y Neurocirugía (INNN), Insurgentes Sur 3877, 14269 Mexico City, DF, Mexico; ^2^Neurochemistry Unit, Instituto Nacional de Neurología y Neurocirugía (INNN), Insurgentes Sur 3877, 14269 Mexico City, DF, Mexico

## Abstract

Multiple sclerosis (MS) is an inflammatory, demyelinating, and neurodegenerative disease of the central nervous system. The hallmark to MS is the demyelinated plaque, which consists of a well-demarcated hypocellular area characterized by the loss of myelin, the formation of astrocytic scars, and the mononuclear cell infiltrates concentrated in perivascular spaces composed of T cells, B lymphocytes, plasma cells, and macrophages. Activation of resident cells initiates an inflammatory cascade, leading to tissue destruction, demyelination, and neurological deficit. The immunological phenomena that lead to the activation of autoreactive T cells to myelin sheath components are the result of multiple and complex interactions between environment and genetic background conferring individual susceptibility. Within the CNS, an increase of TLR expression during MS is observed, even in the absence of any apparent microbial involvement. In the present review, we focus on the role of the innate immune system, the first line of defense of the organism, as promoter and mediator of cross reactions that generate molecular mimicry triggering the inflammatory response through an adaptive cytotoxic response in MS.

## 1. Introduction

Multiple sclerosis (MS) is probably the most enigmatic disease whose etiology remains in controversy. Although its etiology consists in a chronic autoimmune-mediated disease of the central nervous system (CNS) characterized by recurrent episodes of demyelination and axonal lesion, the main pathological characteristic is the “MS plaque” that is unique and different from that seen in other inflammatory diseases [1]. The pathological features of MS plaques include blood brain barrier (BBB) leakage, destruction of myelin sheaths, oligodendrocyte damage, and cell death, as well as axonal damage and loss, glial scar formation, and presence of inflammatory infiltrates [2]. These infiltrates mainly consist of autoreactive lymphocyte T cells, macrophages, microglial cells, ependymal cells, astrocytes, and mast cells, which have the capacity to enter the CNS and incite a proinflammatory reaction, resulting in local tissue injury [3–5]. MS has been recognized as a disease mediated by adaptive immune system where T cells specifically recognizing myelin fragments induce tissue damage and contribute to lesion evolvement [6]. 

Most studies agree that the chronic production of innate immune proteins and the presence of cells of the adaptive immune system in the central nervous system environment could play an essential role to induce neurodegenerative disorders [7]. Although the status of the innate immune system and its relationship to the stages of MS is not well understood, it has been proposed that components of the innate immune system are involved in several deleterious steps in the autoimmune cascade, including activation of myelin-reactive T lymphocytes by antigen presenting cells (APCs) and the development of membrane attack complexes in the CNS; furthermore, in MS patients it has founded inflammatory lesions within the CNS surrounded by infiltrating T lymphocytes, monocytes, and macrophages, as well as activated microglia and reactive astrocytes, suggesting that the innate immune system plays a crucial role in mediating neuronal damage [8]. 

## 2. Experimental Autoimmune Encephalomyelitis Model

The experimental autoimmune encephalomyelitis (EAE) was developed as murine model to clarify the origin of “neuroparalytic accident,” a feared and common complication of vaccination against rabies virus. EAE is a complex condition in which the interaction between a variety of immunopathological and neuropathological mechanisms leads to an approximation of the key pathological features of MS: inflammation, demyelination, axonal loss, and gliosis. Moreover, EAE is often used as a model of cell-mediated organ-specific autoimmune conditions in general. EAE has a complex neuropharmacology, and many of the drugs that are in current or imminent use in MS have been developed, tested, or validated on the basis of EAE studies [9]. This model has allowed identification of the important molecules that drive immunological response in EAE. Some of them are the discovery of ROR-g (RORC) as a master transcription factor for Th17 cell development [10], the identification of the aryl hydrocarbon receptor (AHR) as an essential component in the development of both regulatory T cells (Treg) and Th17 responses [11], and the differential role of the related molecules IL-12 and IL-23 in the susceptibility to autoimmune demyelination [12–14]. 

Actually, it has been induced in a variety of rodents and monkeys, providing models of acute monophasic, relapsing-remitting, and chronic progressive CNS inflammation. The more efficacious models use myelin basic protein (MBP), proteolipid protein (PLP), and myelin oligodendroglial glycoprotein (MOG) as antigenic components of myelin sheath to induce the disease in naive host (mainly nonhuman primates, larger rodents, and mice). The major feature of this model is that histopathology of EAE resembles that of MS [15].

In the 80s decade early studies in EAE demonstrated the role of T lymphocytes in the pathogenesis of MS emerging the TH1 paradigm and supported the evidence that it was founded in MS patients, where TH1 cell induction is associated with a worsening of symptoms; the main evidence for this belief is that relapses tend to be preceded by an increase in the number of circulating IFN-*γ*-secreting T cells [16], in which TH1 cells secreting IFN-*γ* has high capacity to activate macrophages inducing MHC antigens and promoting cell homing [17] and those cells were accumulated in brain lesions from mice with EAE and MS patients [18]. The exogenous administration of IFN-*γ*, increase the exacerbations in MS patients during treatment [19]. And more recently adoptive transfer of autoreactive CD4 T cells has been used as a model to induce EAE [20, 21]. However, both the purely Th1 origin of the pathology in EAE and the extent of the similarity between MS and EAE remain debatable. MS is a very complex disease in which there are many other receptors and cell types involved in the pathogenesis of the disease.

## 3. Leukocyte Endothelial Crosstalk at the Blood Brain Barrier in Multiple Sclerosis

The formation of focal inflammatory lesions within the CNS is a crucial and integral component of the innate immune system on relapsing-remitting MS. These processes are comprised of perivascular, particularly perivenular, cuffs consisting mainly of T lymphocytes and monocyte/macrophages, besides dendritic cells (DCs) and B cells [22]. The migration of these cells represents a key stage in the natural history of the MS disease, but what initiates this event remains unclear.

Our understanding of leukocyte migration has been further complicated by the reemergence of the notion that leukocytes can transmigrate through the body of the endothelial cells (ECs) via pore formation or a phagocytic-like process (transcellular diapedesis) [23] as well as through the EC cell-cell junction (paracellular diapedesis) [24]. Under normal conditions antigen-activated lymphocytes are capable of low-level surveillance throughout the CNS and this limited entry is regulated not by the presence of a vascular barrier but largely by the restricted expression of endothelial cytokine-induced adhesion molecules (CAMs) required for leukocyte capture from the blood [25].

The EC may in turn become activated in response to leucocyte engagement or to leucocyte-derived cytokines such as tumor necrosis factor-alpha (TNF-*α*), interferon-gamma (IFN*γ*), interleukin (IL)-17, IL-22, and IL-1b, which induce or upregulate CAM expression and hence further recruitment of leucocytes leading to an escalation of the inflammatory cascade. Indeed, in EAE and MS the immunoglobulin superfamily (IgSF), molecules intercellular CAM-1 (ICAM-1/CD54), vascular CAM-1 (VCAM-1/CD106) and activated leucocyte CAM (ALCAM/CD166) are all upregulated by vascular endothelium [26–28]. How these events unfold during the initiating phase is not entirely clear; but leucocyte recruitment is undoubtedly of fundamental importance and continues to be a predominant feature during the active life of the lesion.

Accordingly, the first stage of recruitment involves overcoming the shear forces imparted by blood flow and entails the temporary capture of circulating leucocytes through cell-cell interactions mediated by cell surface molecules. In most tissues this initial step is performed by L-selectin, expressed on the majority of leucocytes, and E- and P-selectin on activated ECs. These selectins bind to glycosylated ligands, such as P-selectin glycoprotein ligand 1 (PSGL-1), and mediate the early stage of recruitment characterized by the formation of transient associations (tethering) resulting in leucocyte rolling along the vessel wall in the direction of flow. 

Migration of autoaggressive T cells across the BBB is critically involved in the initiation of experimental autoimmune encephalomyelitis (EAE). The direct involvement of chemokines in this process suggested promotion of G-protein-mediated signaling and adhesion strengthening of encephalitogenic T cells on BBB endothelium *in vivo* [29]. Expression of the lymphoid chemokines CCL19/ELC and CCL21/SLC appears to play an important role during neuroinflammation [30]. Regulation of lymphocyte homing involves secondary lymphoid tissue which leads to T lymphocyte migration into the immunoprivileged central nervous system during immunosurveillance and chronic inflammation [29]. Under homeostatic conditions CCL19 is expressed at the BBB in human and mice and is upregulated during the course of MS and EAE. CCL19 may mediate the activation of T cells and antigen presenting cells expressing the receptor CCR7 [31]. Moreover, human brain EC *in vitro* expresses particularly high levels of CXCL10 and CXCL8; they may contribute to the predominant Th1-type inflammatory response in MS [32].

## 4. Inflammatory Response in Multiple Sclerosis

Four different patterns of pathology with resulting demyelination have been identified in MS lesions: type I is T cell mediated where demyelination is induced by macrophages either directly or by macrophage toxins. While Type II involves both T cells and antibodies, and it is the most common pathology observed in MS lesions; in this case, demyelination is caused by specific antibodies and complement. Type III is related to distal oligodendropathy, where degenerative changes occur in distal processes and followed by apoptosis. Type IV results in primary oligodendrocyte damage followed by secondary demyelination [33].

## 5. Role of Innate Immunity in the Pathology of Multiple Sclerosis

A number of observations have challenged the concept of an “autoimmune reaction” against myelin and adaptive immune response to self-antigens as an integral etiological explanation [35]. These pieces of evidence include the following: (1) pathological studies of the early events in MS show loss of both oligodendrocyte and myelin and the absence of T cells and B cells, suggesting that MS is a process where “other than cell mediated immunity” might be involved [36–38]; (2) large areas of myelin loss are seen in pyramidal and sensorial pathways ascribing this damage to the participation of infiltrating immune cells [39–41]; (3) in some patients with oligodendrogliopathy type III and in rarer cases of Balo's concentric sclerosis, have been found that the demyelinating lesions show T and B cells infiltrates; and finally, (4) in MS patients who received autologous bone marrow transplantation have been found expanding demyelinating lesions with little or no T cells, suggesting that expansion of these lesions is driven by an intrinsic pathological processes within the CNS [42]. An interesting question is why immune response is only focalized in specific plaques and not over all white matter.

According to the evidence, the innate immune system plays an important role in both the initiation and the progression of MS, activating the effector function of T and B cells similar to that process in which a pathogen is involved [43–45]. Although toll-like receptor (TLR) ligands are generally restricted to induce class-switch DNA recombination in T cell-dependent and T cell-independent antibody responses to microbial pathogens [46], they have also been ascribed as causative roles in autoimmune diseases such as EAE and MS. Specific roles of TLRs have been found in EAE [47, 48] and in MS brain lesions [49]; they act as several endogenous ligands capable of inducing TLR signaling, leading to autoimmune neurological diseases.

TLR is a family of immune system receptors localized either in the cell surface or in endosomes of several cell types, in both nonimmune and immune cells, where activation through TLR is given most notably by macrophages and other APCs such as DCs [50] and B cells [51]. Also, TLR are involved in the recognition of pathogen-associated molecular patterns (PAMPs) leading to the transcriptional activation of genes encoding for proinflammatory cytokines, chemokines and costimulatory molecules which subsequently trigger innate immune responses and prime antigen-specific adaptive immunity [52, 53]. TLR1, 2, 4, 6, and 10 are expressed on the cell surface and have been shown to detect and respond primarily to bacterial surface associated to PAMPs, while TLR3, 7, 8, and 9 are located in endosomes of immune system cells and are able to recognize specific nucleic acid (both of DNA or RNA) based on PAMPs [54].

TLRs also have potent functions outside the immune system. Toll and TLR have diverse roles in axonal path finding, dorsoventral patterning, and cell-fate determination [55]. In particular, TLR ligands inhibit the differentiation of several cell types; for example, TLR2 ligands are capable of blocking differentiation of mesenchymal stems cells into osteogenic, adipogenic, and chondrogenic cells [56]; besides, TLR2 and TLR4 differentially regulate hippocampal neurogenesis by unknown ligand(s) [57]. Although, TLR2 is not directly involved in the inflammatory process, its main role described in MS is through the regulation of remyelination. Sloane et al. 2010 demonstrate that TLR2 is expressed in oligodendrocytes and is upregulated in MS lesions. Additionally, TLR2 has the same function as the hyaluronan receptor which is capable of mediating the repressive effects of maturation and remyelination in oligodendrocyte precursor cells (OPCs) [58]. Increased levels of hyaluronan are observed in both EAE lesions and in areas of complete demyelination in MS, perhaps due to altered hyaluronan synthesis, partial hyaluronan degradation, or particular stimulation of TLR2 on oligodendrocytes, events necessary to perform an adequate remyelination blockade [59, 60]. 

On the other hand, TLR9 was identified in B cells and plasmacytoid dendritic cells (pDCs), and unmethylated CpGDNA was identified as a TLR9 agonist [61]. Human TLR9 is only found on pDCs and recognizes viral DNA within the early endosomes at the initial phase of viral infection [62]. It has been associated with the participation of both TLR2 and TLR9 as response to several human viruses infection, including herpes simplex viruses (types 1 and 2), cytomegalovirus, hepatitis C, Epstein-Barr, and varicella-zoster virus [63–65]. Activation of TLR2 is followed by the production of inflammatory cytokines, including IL-6, IL-8, and TNF-*α*. In addition, the induction of IFN-*α* by HSV involves TLR9 [66].

During the pathogenesis of MS, TLR9 is able to recognize DNA within the early endosomes at the initial phase of viral infection. Some studies have shown that TLR9 is capable of driving autoimmunity under different conditions; for example, in mice where TLR9 was deleted from the radio-resistant compartment, such as CNS, mice developed EAE delayed kinetics and severity [67]. Current data on patients confirm results obtained in EAE models, suggesting that TLR9 and MyD88 modulate autoimmune process during the effector phase of the disease and that endogenous “danger signals” can modulate disease pathogenesis [68]. Besides, the activation of APCs via TLR-9 and CD40, events that are likely to occur during the course of an infection, reverses tolerance against myelin antigens and breaks resistance to EAE [67]. Furthermore, regulatory roles in EAE severity have been proposed for TLR4 and TLR9 through altered IL-6, IL-17, and IL-23 levels [48, 69].

On the other hand, TLR3 signaling is capable of suppressing relapsing demyelination in EAE. TLR3 is considered of key importance to antiviral host-defense responses; in EAE the stimulation of TLR3 with polycytidylic acid suppresses relapsing demyelination [70]. Moreover, TLR3 triggers neuroprotective responses in astrocytes, while in controls it induces the growth of axons and neuronal progenitor cells, suggesting additional roles for TLR3-mediated signaling in the CNS and MS [71]. TLR7 and TLR9 appear to upregulate disease severity, since at late stage of EAE, TLR7 and TLR9 mRNA expression is further increased, also suggesting that signaling through these receptors is involved in late active lesions. Moreover, the common TLR adaptor molecule MyD88 is necessary for induction of EAE [72]. Overall, these data suggest that MyD88-dependent signaling through TLR2, TLR4, TLR7, and TLR9 mediates MS progression, while TLR3 activation protects from disease by activation of innate immunity [68].

**Figure 1:**
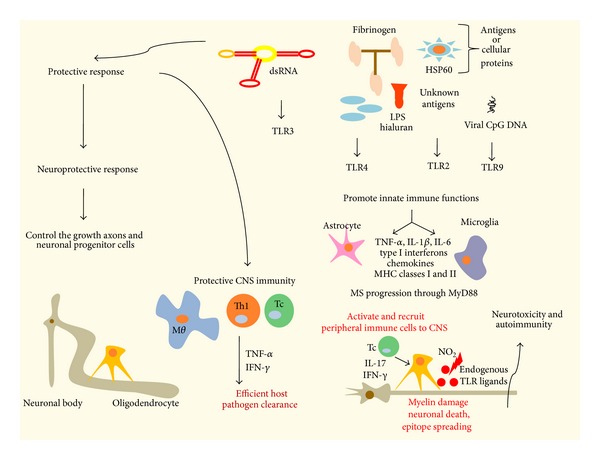
Role of innate immunity in multiple sclerosis. Glial cells, astrocytes, and microglia express a wide variety of TLRs. Stimulation with TLR ligands, dsRNA (TLR3), LPS (TLR4), peptidoglycans (PGN; TLR2 with TLR1/6), and viral CpG DNA (TLR9), promotes an array of immune functions in glial cells, including the secretion of proinflammatory cytokines, chemokines, type I interferons (IFN-*α*/*β*), and an increase in MHC classes I and II expression. TLRs activate macrophages, microglia, and dendritic cells (DCs) resulting in the production of cytokines of the innate immune system such as IL-6, IL-1*β*, and TNF*α*. These cytokines participate in blood brain barrier disruption and lymphocyte attraction to sites of inflammation, promote inflammation, and modulate adaptive immunity. For instance, IL-6 promotes Th17 and B cell differentiation. Th17 and Th1 cells and inflammation will contribute to tissue damage. Finally, MG, microglia, and DC also secrete IFN*β* which, among other immunomodulatory functions, prevents leukocyte adhesion and extravasation across the blood brain barrier. Modified by Carpentier et al. [34].

In addition, TLR7 has been also recently implicated in autoantibody-mediated diseases such as MS. TLR7 is capable of stimulating the maturation and differentiation status of B lymphocytes into immunoglobulin (Ig) secreting cells. Recent finding has shown deficient TLR7-induced IgM and IgG production in MS patients; this might correlate with worsening of disease or impaired immune responses against infections with TLR7-recognized RNA viruses [73, 74]. The modulation of TLR7 could be a potential therapeutic because this is capable of blocking the humoral profile of the disease (Figure 1).

Epidemiological data of developed countries emphasizes the exponential growth on prevalence of autoimmune diseases; this is a plausible consequence of the relative diminution of hygiene conditions and vaccination, implicating a lower contact with pathogens and immune challenges during early life. During neonatal mice stage treated with LPS, it is possible that a high microbial exposure expands antigen repertories and enhances tolerance, delaying the onset and diminishing the severity in EAE. LPS interacts through TLR4 and it seems that in early life it promotes changes in APCs, as lower surface MHCII, CD83 and higher CD80/CD86 costimulatory molecules which elicit migration of Treg cells and expression of immunoregulatory cytokines such as IL-10 under inflammatory conditions of the CNS. Also, splenic APCs from LPS-exposed animals induce less T cell proliferation and selective differentiation of FoxP3^+^ phenotype in response to MOG [75].

## 6. NOD-Like Receptor and Regulation on Multiple Sclerosis

In addition to TLRs, in the past decade a new class of pattern-recognition molecules known as the NOD-like receptors (nucleotide-binding domain, leucine-rich repeat containing family) (NLRs) family of molecules was discovered [76]. Similar to TLRs, NLRs also recognize pathogen-derived molecules and are involved in the first line of defense during infection [77, 78], they can recognize both pathogen- and danger-associated molecular patterns being important sensors of cellular stress that results from infection and cellular instability [79–82], but in contrast to TLR, NLRs sense diverse signals such as reactive oxygen radicals, ultraviolet B (UVB), and low intracellular K^+^ [83] within the host cytosol [84]. NLR proteins NLRP3, NLRP1, and NLRC4 as well as a recently identified HIN-200 protein absent in melanoma 2 (AIM2) are activated by pathogen- and danger-associated molecular patterns (PAMPs and DAMPs, resp.) results in the recruitment of the inflammasome-adaptor protein, ASC (also known as PYCARD), and procaspase-1 [66].

The inflammasome was characterized in 2002 as a cytoplasmic caspase-1 activating, self-oligomerizing signaling complex greater than 700 kDa [85]. Three types of inflammasomes have been identified till date depending on the type of NLR protein involved in its assembly. The NLR expression occurs in macrophages, monocytes, DCs, neutrophils, and cerebral endothelial cells, in the same way occurring either in the membranes of nucleus or cytoplasm, or in the secreted form in granulocytes, monocytes (very low levels), and B and T cells. The NLRP1 inflammasome is composed of NLRP1, ASC, the cysteine proteases caspase-1, and caspase-5 (in mice); the second kind, NLRP2/3 inflammasomes contain NLRP2 or NLRP3, CARDINAL, ASC, and caspase-1 [86] and the third kind of inflammasomes consists of NLRC4 and caspase-1 [87]. NLRP1 and NLRP3 inflammasomes are expressed in lymphocytes T and B; NLRP1 inflammasome is also expressed in motor neurons and cortical neurons [88], and at very high levels in pyramidal neurons and oligodendrocytes, but not in microglial cells or astrocytes [87].

DAMPs are host-derived danger signals released during cell damage or metabolic stress. Factors that trigger inflammasome activation include the mammalian cytosolic ds-RNA, low intracellular K^+^ [83], heat shock proteins (HSP) Hsp60, Hsp70, Hsp90, and gp96; exogenous stress-inducing agents (asbestos, silica, and alum), endogenous instigators of cellular and metabolic distress (ATP, uric acid, fibronectin and mitochondrial dysfunction), and obesity-related factors (fatty acids, lysosomal stress, ceramides, reactive oxygen species (ROS), and hyperglycemia) [83, 85, 86, 89–92]. These multiprotein complexes mediate the proximity-induced autoactivation of caspase-1. Active caspase-1 subsequently cleaves pro-IL-1*β* and pro-IL-18, which is required for their secretion and inflammatory properties. In addition to tightly controlling the activation of IL-1*β* and IL-18, inflammasome signaling can also influence other important biological processes including autophagy and cell death [93]. 

IL-1*β* and IL-18 are produced rapidly under infection, trauma, and stress or as consequence of virus reactivation in the CNS. Both these cytokines share similar structure, activation mechanism, organization of receptor complex, signal transduction pathways, and proinflammatory effects [94] inducing changes in the BBB that bring as a consequence the BBB permeabilization influencing the transport of substances and infiltrating immune cells into the brain from systemic circulation adding onto the neurotoxic effect by a delayed secretion of IL-1 cytokines [86]. The IL-1*β* and IL-18 capture and recognition by the specific receptors IL-1R and IL-18R respectively, induce a signaling cascade via expression of nuclear factor kappa B (NF-*κ*B) and mitogen-activated protein kinase (MAPK), multiple genes encoding inflammatory molecules, namely, CXC-chemokine ligand 8, CX3CL1, IL-6, TNF, endothelial cell selecting (E-selecting), get transcribed [95]. And in hippocampal neurons IL-1*β* as endogenous progeny activates the p38 MAPK signaling pathway and the transcription factor cAMP response element binding protein (CREB) and only NF-*κ*B pathway in hippocampal astrocytes [96], also IL-1*β* is capable of activating resident immune cells and endogenous glial cells [97]. Otherwise IL-18 induces up-regulation of adhesion molecules and stimulates natural killer cell activity [98].

Studies about the inflammasome activation in MS are scarce, but clinical studies have identified an important role for inflammasome-derived cytokines in MS disease pathogenesis. For instance, IL-1*β* and IL-1R antagonist gene polymorphisms were shown to be associated with MS disease severity [99, 100], with main predisposition to develop MS in patients with high ratio of IL-1*β* relative to the naturally occurring IL-1R antagonist and elevated expression of caspase-1, that also it has been observed in MS lesions [101]; besides, caspase-1 and IL-1R are required for the development of EAE [102, 103].

**Figure 2:**
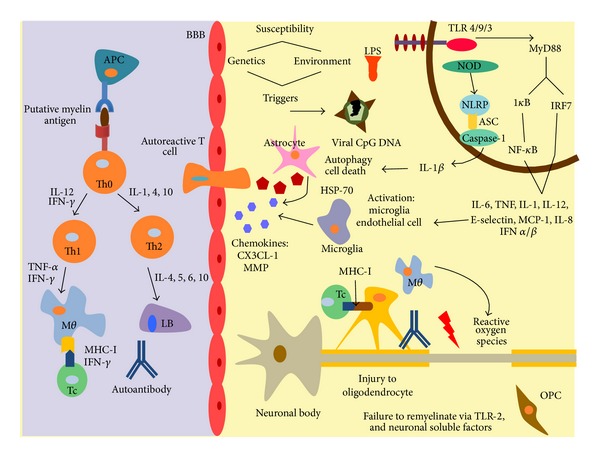
Multiple sclerosis pathophysiology. Contact in early childhood with a pathogen plus other susceptibility factors as racial and demographic background can elicit their reactivation, triggering innate mechanism of defense as toll-like receptors (TLRs), that signalizes downstream through MyD88 (myeloid differentiation primary response 88), and phosphorylated I*κ*B which permits translocation of NF-*κ*B and the transcription of IL-6, TNF, IL-1, IL-12, E-selectin, MCP-1, and IL-8. TLR through IRF7 (Interferon regulatory factor 7) gives the signal to the transcription of IFN *α*/*β*. Another important signal is given by NOD receptors (nucleotide-binding oligomerization domain) activated also by potassium efflux-inducing agents such as ATP and TLR stimulation; PAMS, toxins, danger or stress triggers induce the inflammasome via NLRP that form a complex with ASC (apoptosis-associated speck-like protein containing a CARD) and caspase-1, activating IL-1B, a major factor inducing inflammation, autophagy and cell death, particularly necrosis. All these proinflammatory soluble factors activate microglia and endothelial cells, upregulating expression of adhesion molecules as E-selectin, facilitating the migration of T cells into the SNC. Matrix metalloproteinases (MMP) degrades BBB (blood brain barrier) enhancing further migration of autoreactive T cells and macrophages via chemokines (CX3CL-1). The Th1 response evocated via IL-12 and IFN-*γ* further activates macrophages that in turn do so to T cells CD8+. Th2 response via IL-6 mainly stimulates maturation of B cells and production of autoantibodies. Cytotoxic damage to the oligodendrocyte mediated myelin loss and exposure of the axon to reactive oxygen species, slowing or blocking action potentials and the production of neurological spectrum. There are intents to remyelinate these lesions via OPCs (oligodendrocyte precursor cells), but neuronal factors such as LINGO-1 or TLR2 inhibit their migration.

One of the most effective treatments in relapsing-remitting MS to date is IFN-*β* administration that has been used for more than 15 years as a first-line treatment for MS and its efficacy was demonstrated in the setting of EAE [104]. IL-18 is linked to raised IFN-*γ* in MS patients induced by activated CD4(+) T cells via CD40-CD40 ligand interactions [105]. A recent report has suggested that IFN-*β* attenuates the course and severity of MS by regulating inflammasome activation and subsequent IL-1 production [106]; they found that type 1 interferon potently repressed the activity of the NLRP1 and NLRP3 inflammasomes, thereby suppressing caspase-1 dependent IL-1*β* secretion in mice and MS patients; the inhibitory effect of IFN-*β* is mediated by innate immune cells, such as macrophages and DCs, which inhibit T helper 17 (TH17) responses through interleukin-27 (IL-27) [107, 108] (Figure 2).

## 7. Heat Shock Proteins and Multiple Sclerosis

The HSPs are a group of phylogenetically conserved proteins, which their extracellular expression exerts immunomodulatory functions upon a stress stimulus (i.e., nutrient deprivation, irradiation, hypoxia, heavy metals, oxidative and toxic stress, infections, and exposure to inflammatory cytokines). They are named according to molecular weight, with six families identify, HSP100, HSP90, HSP70, HSP60, HSP40, and the small HSP. The relevance of these molecules relays on being the most abundant soluble intracellular molecules and upon their release; besides, it is given a strong and unequivocal signal of cell death, particularly, necrosis, acting like a danger signal to prevent further cell death by degrading unstable and misfolded proteins. As a recent focus of interest, HSPs enhance immune responses through chaperone activity, arising HSP-antigen complexes, allowing activation of MHC class I stimulated by cross-priming cytotoxic T lymphocytes. This process elicits the maturation of APC and promotes the presentation of unrelated antigens [109]. In the innate immune system, HSPs act as immune-stimulators, like PAMPs, which are recognized by TLRs; this suggested that HSPs can develop autoimmunity response after cell damage [110].

In the brain tissue from MS patients, it has been detected the presence of HSP70-myelin Basic Protein (HSP70-BMP) [111, 112], and HSP70-PLP complexes, both putative antigens with highly encephalitogenicity potential demonstrated in EAE models [113]. Alternative mechanism of innate immune response triggering by HSPs is stimulating the maturation of professional APCs through interaction with the TLR-2, TLR-4, and CD40; these complexes stimulate specific CD4+ T cell responses following the activation of immune system via MHC class II molecules [72].

An other HSP that could play a crucial role in triggering the immune response is Hsp70, which acts as a chemoattractant that elicits the cytolytic effects of NK cell by mediating the interaction with CD94. Released Hsp70 leads to the activation of the NF-*κ*B transcription factor on monocytes, macrophages, and dendritic cells; the activation leads to induction of: (1) proinflammatory cytokine production (IL-12, IL-1*β*, IL-6, TNF, and GM-CSF), (2) chemokine secretion (MCP-1, RANTES, and MIP-1*α*), (3) nitric oxide production (NO) by macrophages (4) enhances the expression of CD83, CD86, and CD40, as well as MHC class II on DC and the migration of these cells to draining lymph nodes, priming adaptive immune responses [114]. All those findings give us evidence that HSPs could drive the switch between the “initial event innate immune response and the perpetuation” adaptive immune response in MS.

## 8. NK Cells in Multiple Sclerosis

Although the evidence suggested that NK cells might play a role in the regulation of MS and EAE, the importance of NK cells to immune regulation remains unclear. Some studies suggest that NK cells enhance the MS due to cytolytic activity, cytokine production, interaction with APCs and T and B cells, while another study indicates that blockade of NK cell homing to the CNS results in disease exacerbation [115, 116]. In EAE, it was found that depletion of NK cells in C57BL/6 mice treatment with a monoclonal antibody (mAb) against NK 1.1 resulted in an increased severity and relapsing pattern of disease [117]. The disease enhancement was associated with increase of T cell proliferation and production of Th1 cytokines in response to the MOG35-55 peptide which induces a mild form of monophasic EAE [118]. 

NK cell homing to the CNS via germ-line deletion of the chemokine fractalkine receptor CX3CR1 resulted in fatal CNS inflammation and demyelination due to inhibition of inflammatory Th17 cells [119]; besides, NK cells exerted direct cytotoxic effect on newly stimulated myelin antigen-specific, encephalitogenic T cells, as well as OVA-specific T cells and Concanavalin A (ConA) stimulated T cells. However NK cells are capable of regulating EAE through killing of syngeneic T cells which include myelin antigen-specific, encephalitogenic T cells and thus ameliorate symptoms [120]. NK cells are an important regulator for EAE in both induction and effector phases. In contrast, it has suggested that NK cells exacerbate MS/EAE. It has been associated with the increase of NK cell activity with higher risk of developing active lesions in relapsing-remitting MS patients. During remission, NK cells predominantly produce IL-5; this is indicative that the NK cells share some properties with Th2 cells and suggested that they are capable of competing with pathogenic autoimmune Th1 cells. Furthermore, in the same cohort of patient higher level of CD95 molecule on the cell surface was found; the authors suggest that it is possible that soluble CD95 might play some role in protection against the CD95-mediated death of NK cells in MS. Interestingly, during relapse of MS, the NK cell expression of IL-5 mRNA and CD95 was significantly reduced. According to this data is interesting to speculate that the functional change of NK cells may play a key role in triggering clinical exacerbation of MS and it is not associated with autoimmune T response [121].

On the other hand, genome wide association studies (GWAS) have identified a number of potential genes associated with MS including receptors for IL-7 (IL-7RA) and IL-2 (IL-2RA); besides, IL-2 and IL-7 pathways have previously been demonstrated to regulate autoimmunity and EAE in animal models [122]. IL2 has been associated with regulation of T-cell proliferation, survival, and differentiation of effectors (Th1/Th2) besides; the function of IL-2 consists of maintaining peripheral T-cell tolerance, and the impairment of regulatory T cells is thought to be the underlying cause of autoimmunity in the absence of IL-2 [123]. IL-2 receptors and have potential suppressive functions, such as natural killer T cells, CD8+ T cells, and CD4+CD25+ regulatory T cells, which might also be altered by IL-2 or anti-IL-2 mAb based therapies.

IL-7/IL-7R signaling is crucial for proliferation and survival of T lymphocytes in humans and in animal models [124]; in humans, IL-7Ra deficiency results in the absence of T cells, but B cell counts remain normal, while in mice the lack of IL-7Ra is essentially devoid of T and B cells. Some studies show that high levels of serum IL-7 predict clinical responsiveness in MS patients undergoing IFN-b therapy. When high IL-7 levels are paired with low IL-17F levels in serum, the prediction is stronger. IL-7 alone or in combination with IL-12 can promote human and mouse T helper 1 (Th1) cell differentiation. These results are consistent with the notion that IL-7 drives a Th1 form of MS, which was previously shown to respond better to IFN-b therapy than the TH17 form of MS [125]. In addition, Axtell et al. show that IL-7Ra–blocking antibodies given to EAE mice before or after onset of paralysis reduced clinical signs of EAE without affecting regulatory T (Treg), B, or NK cells [126]. Therefore, blockade of IL-7 or IL-7Ra may be a potential therapeutic strategy for treating MS.

## 9. Neutrophils in Multiple Sclerosis

Neutrophils are essential to contain and clear infectious agents, but due to their indiscriminate histotoxic potential they are tightly regulated by a mechanism that involves “priming” before full activation [127]. Neutrophils can be primed by a wide range of molecules including proinflammatory cytokines such as TNF*α*, platelet activation factor (PAF), IFN-*γ*, granulocyte-macrophages colony stimulation factor (GM-CSF), IL-6, or IL-8 that can modify neutrophil life span [128, 129]. Patients with relapsing-remitting multiple sclerosis (RRMS) have an increased number of neutrophils that regulated phenotypic changes such as reduction of apoptosis and higher expression of TLR2, FPR1, CXCR1, and CD43 [130]. Enhanced neutrophil activation during infection in RRMS patients exacerbates and prolongs inflammation that might explain an association between infection and relapses of MS.

In patients with RRMS in relapse, was found a correlation between high neutrophil count and up-regulation of GCS and CXCR1 as well as an inhibition of apoptosis and induction of inflammatory response. Also, these patients present an increase of IL-8; this cytokine prolongs the neutrophil survival [111]. Neutrophils RRMS patients are not only more abundant but also express higher levels of TLR2, CD43, FPR1, and CXCR1, which support the hypothesis that neutrophils in RRMS are primed by the chronic inflammatory milieu, as these receptors are upregulated by priming agents [131, 132].

A higher release of granule proteins such as elastase and cathepsin G by primed neutrophils could contribute to MS pathogenesis not only by damaging tissue [133] but also by enhancing T cell activation [134]. Furthermore, neutrophilic granules also contain the matrix metalloproteinase 9 (MMP-9), which participates in BBB impairment and is increased in MS patients during relapse [135].

Not only the number of neutrophils is increased in patients with MS, but also there is an oxidative burst of neutrophils from RRMS patients after *in vitro* stimulation with Formyl-Methionyl-Leucyl-Phenylalanine (fMLP) [136]. The enhanced oxidative burst could contribute to demyelination and tissue injury in MS, although a paradoxical protective role for oxygen species has been suggested [130, 137].

## 10. Mast Cells

Mast cells (MCs) are components of the innate immune system arising from multipotent hematopoietic progenitors cells and are phenotypically identified for high expression on their surface of the tyrosine kinase receptor c-*kit* (CD117) and the high-affinity Fc receptor for IgE (Fc*ε*RI) that is the main characterized mode of MC activation through IgE-mediated immune reaction [138]. The cross-linking of Fc*ε*RI-bound IgE with a multivalent antigen induces aggregation of two or more Fc*ε*RI molecules and activates downstream intracellular-signaling events leading to degranulation and synthesis of new mediators [139].

MC-granules contain biogenic amines (histamine and, only in rodents, serotonin), serglycin proteoglycans (heparin and chondroitin sulphate), serine proteases (tryptases, chymases, and carboxypeptidases), cytokines (such as TNF-*α*), and growth factors (such as vascular endothelial growth factor A (VEGFA)) [140]. Fc*ε*RI-mediated activation of MCs induces also the *ex novo* synthesis of lipid mediators such as prostaglandins (PGD2, PGE2) and leukotrienes (LTB4, LTC4), cytokines (e.g., TGF-*β*, IL-4, and IL-10), chemokines (such as CC-chemokine-ligand 2), and growth factors (e.g., nerve growth factor (NGF)) [141, 142].

MCs in the CNS can be found in perivascular locations more specifically in the leptomeninges forming part of the BBB and in mice. MCs have been identified in perivascular areas of leptomeninges, hippocampus, habenula, and thalamus [143, 144] which has led to speculation of a possible contribution of these cells in regulating the trafficking of immune cells through the BBB [145, 146].

Some pieces of evidence that support the role of the MCs in the pathology of MS are as follows. (1) MCs have been detected within demyelinated lesions, often in perivascular areas associated with immune cell infiltrates, but also in the CNS parenchyma and it is more frequently observed in chronic-active plaques than in acute lesions [146–148]. (2) Myelin proteins such as MBP can activate rat MCs through interaction with scavenger receptors [149, 150]. (3) The concentration of MCs tryptase was found significantly higher also in the cerebrospinal fluid of MS subjects [151]. Recently MC has also been implicated in the development of MS and EAE [152]. However, today the exact role of MCs in CNS autoimmune disease is highly debated. 

## 11. The Mononuclear Phagocyte System 

The mononuclear phagocyte system (MPS) comprises the cell hematopoietic lineage derived from progenitor cells in the bone marrow. These bone marrow myeloid progenitor cells differentiate to form blood circulating monocytes and then upon activation enter tissues to become resident tissue macrophages. There are three main features of macrophages; (1) evidence of endocytic activity and stellate morphology: (2) expression of certain enzymes detected by histochemical staining (esterases and lysosomal hydrolases), and (3) the nonspecific uptake of particles (e.g., latex, colloidal carbon) through endocytic receptors or complement-coated particles [153]. 

Microglia is the principal effector cell of the innate immune system that resides on CNS; it has a central role of initiating the acute inflammatory response and clearance of damage tissue; also, during the phase of scarring, the irreversible injury is associated with residual neurological deficits. During inflammatory disorders, such as MS, monocytes are repeatedly recruited from the periphery, thereby reinforcing the local inflammatory reaction within the CNS. In MS macrophages act as APCs perpetuating epitope spreading upon T cell traffic on brain through BBB [153]. Animal models evidence that inflammatory lesions are composed principally of T cells with neural-antigens specific TCR; however, the activation of APC pathways is required for the maintenance of the continuity of this process, and microglia has this capacity, enhancing homing signals.

In normal white matter there is low basal expression of MHC class II that is upregulated after damage or immune reactions originated in systemic compartments or local environment. Low levels of circulating endotoxins or PAMPs could trigger the expression of MHC class II on the perivascular macrophages, this fact prompt us to hypothesized why viral infections could be related with relapsed patients. The initiation of a competent APC status for microglia set a cascade of infiltrating adaptive immune cells, specifically neural-specific T cells, propagation and resolution of inflammatory lesions [154]. 

## 12. Th17 Immune Response in Multiple Sclerosis

Th17 cells are characterized by the production of a distinct profile of effector cytokines, including IL-17A, IL-17F, IL-6, IL-9, IL-21, IL-22, IL-23, IL-26, and TNF*α* [155, 156]. In addition to CD4+ IL-17+ Th17 cells, a new putative subtype of IL-17 producing CD4+ T cells with CD4+ IL-17+ IFN*γ*+ (Th17-1 cells) also, phenotype, has been identified [157]. While Th17 cells express CCR6 and CCR4, Th17-1 cells express CCR6 and CXCR3 [158, 159]. The central role of Th17-produced cytokines in the brain is the induction of inflammation; therefore, Th17-mediated inflammation is characterized by neutrophil recruitment into the CNS and myelin loss [160, 161]. 

In MS lesions, the presence of high levels of IL-17 induce a strong inflammatory response, that could play an important role in the pathogenesis and exacerbations of the disease [162]. The IL-17-producing T cells (CD4+ or CD8+) have been detected in both acute and chronic MS [163]. In the preclinical stages of the disease, the autoreactive Th17 cells were found in the peripheral blood mononuclear cells (PBMCs) but not in the CNS. Moreover, the amount of Th17 cells was significantly higher in the cerebrospinal fluid (CSF) of RRMS patients during relapse, in comparison with same patients during remission or in patients with other noninflammatory neurological diseases [164]. The amount of production of IL-17 correlates with the number of active plaques as seen on magnetic resonance imaging studies (MRI) and the severity of MS [165, 166].

Infiltrating T cells and glial cells inside of CNS induces production of IL-17 [163] exerting pathogenic function by enhancing the microglia activation; therefore, exposure to microglial cells leads to increased generation of IL-6, MIP-2, nitric oxide, neurotrophic factors, and adhesion molecules. Furthermore, addition of IL-1b and IL-23 enhances the production of IL-17 in microglia [167] and release of matrix metalloproteinase-3 (MMP-3) that disrupts the BBB and enhances the local recruitment of neutrophils to the site of the inflammation. The increase of protease activity allows homing a large number of monocytes and macrophages leading to chronic myelin and axonal damage [168, 169].

## 13. Treatment by Immune Modulators 

Over the last two decades a number of drugs, including immunomodulatory and immunosuppressive agents such as interferon-*β*, glatiramer acetate, and the monoclonal antibodies such as natalizumab and daclizumab have shown beneficial effects in patients with MS. Although these therapies are able to modulate the immune adaptive response, they do not inhibit innate immune cells, such as microglial cells, macrophages, and dendritic cells, that participated in the progression of MS. IFN-*β* is one of several immunomodulatory drugs currently available to treat patients with relapsing-remitting MS [170, 171], displaying significant beneficial effects on disability progression and relapse rate [172]. The mechanism(s) of action of IFN-*β* is clearly complex with demonstrated effects on antigen presentation, costimulatory molecule expression, T-cell proliferation, and leukocyte migration [173].

In conclusion, the autoimmunity mediated by autoreactive T cells activates the innate immune system (epithelial barriers, receptors to pathogen associated patterns like toll-like receptors) as first line of defense of the host, eliciting the immune tolerance. It has been demonstrateet that in so many ways the innate immune system (TLR7, 3, 9, neutrophils, macrophages) offerd the interface between adaptive response, not only regulating the cellular damage, but also allowing that autoreactive CD4+ T cells reacts against the oligodendrocyte. A continuum effort should be given to the inhibition of the initial triggering of the innate immune system. 

## Abbreviations

MS: Multiple sclerosis
BBB: Blood brain barrier
APC: Antigen presenting cells
DCs: Dendritic cells
ECs: Endothelial cells
CAMs: Cytokine-induced adhesion molecules
TNF-*α*: Tumor necrosis factor-alpha
IFN: Interferon
IL: Interleukin
IgSF: Immunoglobulin superfamily
PSGL-1: P-selectin glycoprotein ligand 1
EAE: Experimental autoimmuneencephalomyelitis
TLR: Toll-like receptor
MBP: Myelin basic protein
MOG: Myelin oligodendroglial glycoprotein
PAMPs: Pathogen-associated molecular patterns
OPCs: Oligodendrocyte precursor cells
pDCs: Plasmacytoid dendritic cells
Ig: Immunoglobulin
IgM: Immunoglobulin M
IgG: Immunoglobulin G
NLRs: Nucleotide-binding domain, leucine-rich repeat containing family
ROS: Reactive oxygen species
HSP: Heat shock proteins
E-selectin: Endothelial cell selecting
MAPK: Mitogen-activated protein kinase
CREB: Response element binding protein
NF-*κ*B: Nuclear factor kappa B
mAb: Monoclonal antibody
PAF: Platelet activation factor
GM-CSF: Granulocyte-macrophages colony stimulation factor
RRMS: Relapsing-remitting multiple sclerosis
PBMC: Peripheral blood mononuclear cells
CSF: Cerebrospinal fluid.
